# Eosinophil-related diseases during treatment with glucagon-like peptide one receptor (GLP-1 RA): a case report and review of the literature

**DOI:** 10.1007/s10067-023-06612-w

**Published:** 2023-05-18

**Authors:** Iván Posso-Osorio, Carlos Julio Vargas-Potes, Mauricio Mejía, Carlos A. Cañas

**Affiliations:** 1 Universidad Icesi, Facultad de Ciencias de la Salud, Cali, 760031 Colombia; 2 Unit of Rheumatology, Fundación Valle del Lili, Unidad de Reumatología, Cali, 760031 Colombia; 3 Universidad Icesi, CIRAT: Centro de Investigación en Reumatología, Autoinmunidad y Medicina Traslacional, Cali, 760031 Colombia; 4 Fundación Valle del Lili, Unidad de Radiología, Cali, 760031 Colombia

**Keywords:** Eosinophilic fasciitis, Semaglutide, Adverse event, Side effect

## Abstract

Glucagon-like peptide one-receptor agonists (GLP-1 RA) are drugs that differ in their pharmacological composition and homology to human GLP-1 and are used most frequently for the treatment of type 2 diabetes and weight loss. There are isolated reports of eosinophilic adverse reactions associated with GLP-1 RA. We present the case of a 42-year-old female patient who, after starting weekly subcutaneous semaglutide, developed eosinophilic fasciitis with favorable clinical evolution after the discontinuation of semaglutide and the initiation of immunosuppression. A review of the eosinophilic adverse events that have been previously reported with GLP-1 RA is provided.

## Introduction

As a serendipitous event, the peptide exendin-4 from the saliva of a venomous lizard (*Heloderma suspectum*, the Gila monster) was found to be homologous to mammalian glucagon-like peptide one receptor (GLP-1) and able to bind and activate GLP-1 receptors [1]. Synthetic exendin-4 was named exenatide and, without further modification, was the first GLP-1 receptor agonist (GLP-1 RA) approved to treat type 2 diabetes (T2D) [2]. At present, several GLP-1 RAs have been approved and used clinically for the treatment of diabetes mellitus and certain forms of obesity, including lixisenatide, liraglutide, dulaglutide, albiglutide, and semaglutide [3–5]. The side effects most frequently reported with GLP-1 RAs are nausea, vomiting, and diarrhea [6]; rarely, pancreatitis [7], acute kidney injury [8], or hypoglycemia are reported [9]. Isolated cases of eosinophilic reactions have been reported during treatment with GLP-1 RAs, including peripheral eosinophilia, eosinophil-rich bullous pemphigus, acute interstitial nephritis, eosinophilic panniculitis, and eosinophilic hepatitis, among others [10–28].

Here, we describe the case of a patient who developed eosinophilic fasciitis after initiating semaglutide.

## Case report

A 42-year-old female patient with no pathological history consulted the rheumatology clinic. Six weeks before the consultation, she had initiated subcutaneous semaglutide for weight loss. Two weeks after that, she starts complained of myalgia and edema in the four limbs. The review of symptoms by systems was negative. On physical examination: heart rate 75 bpm, blood pressure 105/70 mmHg, respiratory rate 18 X’. Cardiopulmonary, neurologic, and abdominal physical exam was normal. Musculoskeletal examination showed pitting edema of four limbs. The rest of the joints had no positive findings; in lower limbs, skin induration was evidenced together with the Groove sign (orange peel).

Laboratory test results were as follows: hemoglobin: 13.2 g/dL, leukocytes: 8210 cells/mm3, neutrophils: 4980 cells/mm3, lymphocytes: 1450 cells/mm3, eosinophils: 950 cells/mm3, erythrocyte sedimentation rate: 5 mm/hour; C-reactive protein: 0.38 mg/dL, thyroid-stimulating hormone: 7.66 μIU/mL, free thyroxine: 0.80 ng/dL, thyroid peroxidase antibodies: positive (804 IU/L), vitamin D 25-OH: low (21), rheumatoid factor: negative, citrullinated peptide antibodies (antiCCP): negative, antinuclear antibodies by indirect immunofluorescence: 1:160 titer in a fine granular pattern, extractable nuclear antibodies (ENAs): negative, immunoglobulin G levels: 920 g/L (reference value: 552-1631), creatine phosphokinase: 18 IU/L, creatinine: 0.52 mg/dL, urinalysis: normal, IgE levels: elevated.

**Fig. 1:**
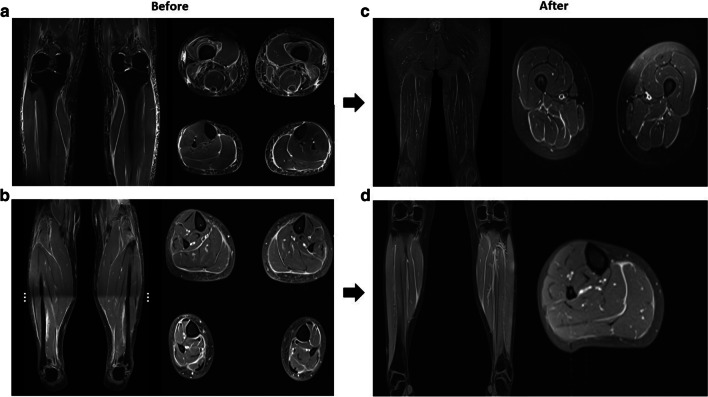
Contrast MRI. **a** Coronal and axial STIR images showing edema of both superficial and deep fascia, subcutaneous fat tissue, and variable edema of different muscle groups. **b** Coronal and axial post gadolinium GRE fat-saturated T1-weighted images showing enhancement of superficial and deep fascia, consistent with active inflammation. **c** (Follow-up at 4 months) Coronal and axial T2 weighted STIR images of distal thighs. Note the significant reduction of edema of deep and superficial fascia, along with disappearance of subcutaneous and muscle edema. **d** (Follow-up at 4 months) Coronal and axial post contrast T1-weighted fat-suppressed GRE images through the upper legs. Note a mild gadolinium uptake of deep and superficial fascia of the posterior compartment.

Given these findings, contrast-enhanced magnetic resonance imaging (MRI) of the lower limbs was performed, which showed diffuse linear thickening, with signal hyperintensity in sequences sensitive to edema and marked enhancement in the postcontrast phase of all the superficial and deep fascia in the muscular compartments of both legs (Fig. 1a and b) were found. For all the reasons mentioned above, a diagnosis of eosinophilic fasciitis was made. and biopsy was not indicated due to high risk of complications. The biopsy was not done due to high risk of complications. This decision was made because a medical board was held with the dermatology, orthopedics, and rheumatology team, where the conclusion was reached not to perform the biopsy due to the severe edema that the patient presented, and a high risk of infection, ulcer development, bleeding, tissue damage, and morbidity in general was considered. The drug Semaglutide was discontinued, and management was started with pulses of methylprednisolone 1 g daily for three days and then oral methylprednisolone 1 mg/kg/day. Subsequent management with IV cyclophosphamide 1000 mg monthly for 4 months was started. The glucocorticoid was progressively reduced. Thyroid hormone replacement was initiated due to diagnosis of primary hypothyroidism.

In the following weeks, the patient presented a total resolution of symptoms, the normalization of eosinophil count, and resolution of four limbs edema. Four months after presentation, a new contrast MRI showed a notable decrease in the intensity and extremities (Fig. 1c and d).

## Discussion

Eosinophilic fasciitis is a rare fibrosing disorder first described in 1974 by Shulman, who described it as a syndrome of “diffuse fasciitis with hypergammaglobulinemia and eosinophilia” [29]. Until the end of the 20th century, eosinophilic fasciitis was usually represented as a variant of scleroderma or a scleroderma-like disorder. However, it is now considered a separate entity [30]. Naschitz et al. classified the disease within the “fasciitis-panniculitis syndromes” group because they share morphological characteristics in the fascia and subcutaneous tissue [31]. The current classification criteria, proposed by Pinal-Fernandez et al. [32] and Jinnin et al. [33], include plaque-like sclerotic lesions on the skin, skin induration, and histopathological and imaging changes related to a thickening of the fascia, eosinophilia, and hypergammaglobulinemia.

This disease is characterized by abrupt onset edema with subsequent induration and thickening of all the extremities (70–83%), with little to minimal involvement of the face or phalanges. Classically, it presents a peau d’orange-like appearance and the “groove sign” (areas of depression along the course of the superficial veins). Due to the infrequent nature of the disease, epidemiological data are primarily based on case reports. A slight predominance in women has been found, with an average age of onset between 20 and 60 years [34].

Whether eosinophilic fasciitis has an autoimmune pathogenesis is not clear since a specific antigen that is the target of a response mediated by T or B lymphocytes has not been found [34]. However, it is considered an inflammatory disease with hypergammaglobulinemia (35–46%), adequate response to steroids (in 97% of patients with monotherapy), slight antinuclear antibody (ANA) positivity (5.7–17%), and eosinophilia in peripheral blood (58–85%) [35], which is often present during the acute phase with reduced levels posttreatment; however, the latter is not a major criterion for the diagnosis [32, 33]. Even though our patients had slight antinuclear antibody (ANA) positivity, they did not have other diagnostic criteria for systemic lupus erythematosus.

The pathophysiology of eosinophilic fasciitis is still not well elucidated. Until now, it has been known that part of this process includes the infiltration of macrophages, eosinophils, and CD8+ lymphocytes in the fascia with the release of cationic protein and granzyme B, which have toxic and potentially fibrogenic properties [36]. There is also an essential role of molecules such as tissue inhibitor of metalloproteinases one (TIMP-1), IL-5, IFN-gamma, and IL-10, with elevated serum levels of soluble CD40 ligand (sCD40L, a marker of activated CD4+ lymphocytes) [37].

Possible factors that have been associated with triggering disease include infections (Borrelia, Mycoplasma), drugs (angiotensin-converting enzyme inhibitors, antituberculosis medicines, phenytoin, simvastatin, atorvastatin, infliximab, pembrolizumab), and radiation [30]. Due to the multiple risk factors that have been described as possible triggers, it is essential to describe new medications that may have an implication in developing this disease. We made an unstructured review of the literature and searched several online databases: PubMed and Google Scholar. MeSH terms linked to the Boolean connectors were used as follows: “Eosinophilia,” “hypereosinophilic syndrome,” “eosinophilic reactions,” “GLP-1,” and “adverse events.” Only articles published in English were included. The relevant references for our review were included. We include articles describing eosinophilia associated with the use of GLP-1 agonist drugs. We reviewed citations, abstracts, and full-text articles and selected 20 papers. Table 1 shows the reported cases of hypereosinophilia and hypereosinophilic syndromes associated with the use of GLP-1 RAs.

**Table 1:** Cases of hypereosinophilia and hypereosinophilic syndromes associated with the use of GLP-1 RAs

No.	Age	Sex	GLP-1 RA	Eosinophil-related adverse effect	Improvement with drug discontinuation	Ref.
1	52	F	Exenatide extended release	Peripheral eosinophilia (24.3%, normal range 1–4%)	Yes	10
2	68	M	Liraglutide	Generalized erythematous rash, peripheral eosinophilia, lymphocyte, and eosinophil infiltration in the papillary dermis	Yes	11
3	75	M	Liraglutide	Bullous pemphigoid, histopathology with subepidermal blister, mixed perivascular infiltrate with numerous eosinophils, and pigment incontinence	Yes	12
4	61	F	Semaglutide	Bullous pemphigoid, subepidermal vesiculation with brisk mixed dermal infiltrate containing many eosinophils	Yes	13
5	83	M	Liraglutide/exenatide	Acute interstitial nephritis, biopsy with diffuse tubulointerstitial infiltration with numerous eosinophils	Yes	14
6	54	M	Exenatide extended release	Eosinophilic panniculitis	Yes	15
7	N/A	F	Liraglutide	Drug-induced liver injury negative for markers of autoimmune hepatitis, massive hepatic necrosis, and extensive eosinophilic infiltration at biopsy	No	16
8	83	F	Semaglutide	Acute kidney injury, interstitial lymphoplasmacytic, and eosinophilic infiltration and evidence of acute tubular injury	No	17
9	30	M	Semaglutide	Acute interstitial nephritis, patchy interstitial inflammatory infiltration (lymphocytes, plasma cells, mild eosinophils)	Yes	18
10	59	M	Exenatide	Nodular, eosinophil-rich granulomatous panniculitis	ND	19
11	58	M	Exenatide	Moderately severe diffuse tubulointerstitial nephritis, abundant eosinophil infiltration	No	20
12	62	M	Exenatide	Eosinophilic sclerosing lipogranuloma	Yes	21
13	84	M	Dulaglutide	Morbilliform drug eruption, interface dermatitis with eosinophils infiltrating in the dermis	Yes	22
14	53	F	Dulaglutide	Autoimmune hepatitis, necroinflammatory activity with mixed infiltrates containing plasma cells and eosinophils	Yes	23
15	38	F	Exenatide extended release	Eosinophil-rich granulomatous panniculitis	ND	24
16	60	M	Exenatide	Septal panniculitis with mononuclear cells and prominent admixed eosinophils	ND	25
17	52	F	Exenatide extended release	Drug-induced eosinophilia pneumonia	Yes	26
18	40	F	Liraglutide	Acute generalized exanthematous pustulosis, intraepidermal neutrophilic abscesses, and perivascular lymphoplasmacytic inflammation with numerous eosinophils	Yes	27
19	52	F	Liraglutide	Hepatotoxicity, peripheral eosinophilia (8.5%, absolute count of 600/μL)	Yes	28
20	42	F	Semaglutide	Eosinophilic fasciitis, peripheral eosinophilia (absolute count of 950/mm^3^), MRI lower limbs: enhancement in the postcontrast phase of all the superficial and deep fascia in the muscular compartments	Yes	Our patient

Abbreviations: *F*, female; *M*, male; *N/A*, not available; *GLP-1RA*, glucagon-like peptide one-receptor agonists; *Ref.*, reference; *MRI*, magnetic resonance imaging

To date, there are no case reports of eosinophilic fasciitis associated with the use of semaglutide; however, according to Naranjo’s score [38], our case reported here constitutes a probable adverse reaction (4 points).

The pathophysiological reasons that would explain this case are not clear. It has been reported that eosinophils have GLP-1 receptors similar to neutrophils and that the GLP-1 receptors on these cells have an attenuating function. In a study published in 2017, it was shown that patients with asthma had lower expression of GLP-1 receptor in eosinophils than healthy controls; additionally, the exposure of patients to GLP-1 analogs significantly decreased the expression of eosinophil surface activation markers after LPS stimulation and reduced eosinophil production of IL-4, IL-8, and IL-13, but not IL-5, which would be in favor of a paradoxical reaction [39]. The clinical evidence of eosinophilic reactions with these type drugs is evidenced in the increasing number of clinical cases that are being reported.

As a limitation of our study, a deep fascia biopsy was not performed. Still, as explained above, a diagnosis was achieved with the least invasive methods for our patient, reducing morbidity and possible complications.

Concerning our patient’s treatment, we decided to discontinue semaglutide and to treat her with pulses of methylprednisolone and intravenous cyclophosphamide due to the severity of the clinical presentation. The patient’s case has presented a favorable evolution.

We report a case of eosinophilic fasciitis associated with semaglutide. The opportunity to investigate the pathophysiology of this event is open to further discussion.
